# Chromosome-Contiguous Reference Genome for *Spirometra* to Underpin Future Discovery Research

**DOI:** 10.3390/ijms26136417

**Published:** 2025-07-03

**Authors:** Neil D. Young, Richard Malik, Alexa Brown, Tao Wang, Amanda Ash, Pasi K. Korhonen, Robin B. Gasser

**Affiliations:** 1 Melbourne Veterinary School, Faculty of Science, The University of Melbourne, Parkville, VIC 3010, Australia; tao.wang1@unimelb.edu.au (T.W.); pasi.korhonen@unimelb.edu.au (P.K.K.); 2 Centre for Veterinary Education, The University of Sydney, Sydney, NSW 2006, Australia; richard.malik@sydney.edu.au; 3 WAVES Veterinary Hospital, 1/640 Beeliar Drive, Success, Perth, WA 6164, Australia; alexaleebrown@gmail.com; 4 School of Medical and Molecular Forensic Sciences, Murdoch University, Perth, WA 6150, Australia; a.ash@murdoch.edu.au

**Keywords:** tapeworm, cestode, *Spirometra*, reference genome, advanced sequencing technology, bioinformatics

## Abstract

Sparganosis is a neglected food- and water-borne zoonotic disease caused by members of the tapeworm genus *Spirometra*. More than 1600 human cases have been reported in the literature, primarily in Korea and China; however, the clinical significance of sparganosis is likely underestimated. The control of this disease is challenging in endemic regions because of the complexity of its lifecycle and the involvement of many animal host species, and treatment of clinical disease in humans and animals with selected drugs (e.g., mebendazole and/or praziquantel), even at elevated doses, is often ineffective, such that novel interventions are needed. It is anticipated that the use of molecular technologies should allow the identification of new intervention targets in crucial biological processes and/or pathways of *Spirometra* spp. While some draft genomes of *Spirometra* have been produced, their assemblies are incomplete. Here, we employed an advanced DNA sequencing–informatic approach to assemble and annotate the first high-quality genome of an isolate of *Spirometra* from Australia, with chromosome-level contiguity and a curated gene set. This improved genome provides a useful resource to support fundamental and applied molecular investigations of *Spirometra* species and should assist in the design of new tools for the intervention against sparganosis of companion animals (including dogs and cats) and humans.

## 1. Introduction

Sparganosis is a food- and water-borne zoonosis caused by a larval tapeworm of the genus *Spirometra* (Cestoda: Diphyllobothriidea). More than 1600 human cases have been reported in the literature, with most originating from Korea and China [1,2]. However, the clinical significance of sparganosis has been neglected, such that the prevalence of this disease is likely underestimated in most countries, given the limited knowledge of its geographic distribution around the world [1,3].

The transmission of sparganosis/*Spirometra* can be complex, involving carnivorous definitive hosts (usually canids and felids harboring the adult tapeworm), a first intermediate host (a copepod), and second intermediate/paratenic hosts (e.g., frogs, snakes, and/or other vertebrates, including humans as accidental hosts). People usually become infected via consuming drinking water contaminated with procercoid-infected copepods or by consuming or employing plerocercoid-infected raw flesh from snakes or frogs or other tetrapods in traditional poultices to treat ailments of the eye or skin [1]. Sparganosis results from plerocercoids (or spargana) that migrate into and/or proliferate in a range of tissues/organs, and symptomology depends on the localization of these larvae in the body. The main clinical forms include (i) subcutaneous sparganosis, caused by plerocercoids in connective tissues and/or superficial musculature (e.g., trunk, limbs, or scrotum), which commonly manifests as slow-growing nodules (0.5–2 cm in size) and/or localized erythema; (ii) cerebral sparganosis, where larvae migrate to the central nervous system (including the cerebral hemispheres, cerebellum, and/or spinal cord) and cause neurological and associated signs; (iii) ocular sparganosis, where larvae migrate into the (sub-)conjunctiva or the orbit and cause irritation, oedema, lacrimation, and/or blindness, but rarely invade the eye; (iv) visceral sparganosis caused by larvae in internal organs or organ systems; and (v) proliferative sparganosis caused by continuously replicating larvae (spargana), which can result in the systemic dissemination of disease in various tissues and organs [1,4,5,6].

The accurate identification of *Spirometra* to species is central to reaching a diagnosis and to tracking this parasite throughout what is a complex transmission pattern. Since conventional histopathological methods cannot accurately identify the disease-causing spargana to the species level, the use of molecular tools has become crucial from an applied perspective, particularly for the diagnosis of disease/infection, and for fundamental epidemiological and systematic investigations [1]. A recent, comprehensive review of the genus *Spirometra* [6] concluded that there are presently four valid species, namely *S. erinaceieuropaei*, *S. decipiens*, *S. folium* and *S. mansoni*, and molecular findings indicate at least six separate lineages, typified by distinct geographical distributions, although the number of species is likely to be considerably higher [6].

Comprehensive genomic investigations should underpin fundamental research on *Spirometra*, facilitating the elucidation of molecular processes and pathways in this parasite genus and/or those involved in the disease pathogenesis, as well as the identification of diagnostic markers and new targets for disease intervention. Draft genomes of larval material from a human patient of Chinese ethnicity with chronic cerebral sparganosis in the UK [7], from BALB/c mice infected with spargana originally isolated from a Venezuelan patient in 1981, and *Spirometra* from a Japanese four-lined ratsnake (*Elaphe quadrivirgata*) in 2014 [8] have provided initial insight into genes, encoded proteins, gene ontology (GO) networks and metabolic pathways, and enabled some comparative studies. The sequencing and assembly of new genomic and transcriptomic resources have been accompanied by the development of new informatic tools and workflows to accelerate both fundamental and applied molecular research of this and related groups of parasitic worms (reviewed in [9,10,11]).

Despite these developments, a limitation for detailed genetic, taxonomic, and molecular studies of members of the genus *Spirometra* has been the fragmented nature of published genomes, assembled using data produced using first- and/or second-generation sequencing methods [7,8]. Long-read and long-range (i.e., third-generation) sequencing technologies and advanced informatic methods are now enabling substantially enhanced genome assemblies [12,13,14,15,16,17], achieving improved scaffold contiguity and gene models and allowing the discovery of novel, structurally-complex and repetitive, non-coding DNA regions not adequately represented in fragmented genomes. In the present study, we employed a combination of in situ Hi-C (chromosome conformation capture technology) and Pacific Biosciences (PacBio) single-molecule real-time (SMRT) sequencing [18,19,20] to generate a chromosome-level genome assembly for a representative of *Spirometra from Australia*.

## 2. Results

### 2.1. Initial Genetic Characterization

**Figure 1:**
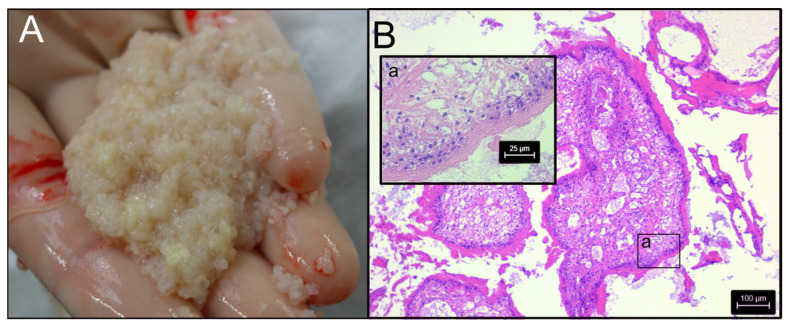
Panel (**A**): Material collected from the abdominal cavity of a dog during exploratory surgery suspected to be proliferative spargana of *Spirometra* (Spiro_Aus1). Panel (**B**): Examination of histological, hematoxylin and eosin-stained sections of these structures by light microscopy (40-times magnification) revealed tegument, musculature and vacuoles (inset-image a), which were indeed consistent with spargana (cf. [21]).

**Figure 2:**
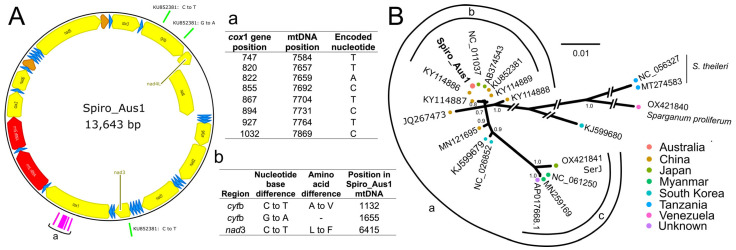
Panel (**A**): Mitochondrial genome representing *Spirometra* (isolate Spiro_Aus1) from a dog with sparganosis in Australia, which was a 99.99978% match to a mitochondrial genomic sequence in GenBank (accession no. KU852381.1) at the nucleotide level. The direction of gene transcription is shown with an arrow. Long (*rrn*L) and short (*rrn*S) ribosomal RNA subunits are shown in red, protein-encoding genes are shown in yellow, and transfer RNAs are shown in blue. Nucleotides used to assign haplotype SH2 [22] are shown in pink and listed in inset-table a; nucleotide positions that were distinct between Spiro_Aus1 and KU852381.1 are shown in green, and the amino acid changes linked to nucleotide alterations are listed in inset-table b. Panel (**B**): Phylogenetic relationship of Spiro_Aus1 with *Spirometra* representatives established following Bayesian inference (BI)-based analysis of aligned, concatenated mitochondrial nucleotide sequence data representing 12 mitochondrial protein-encoding genes. Nodal support is given as a posterior probability (pp). The scale-bar indicates phylogenetic distance (in substitutions per site). There was clear support for three proposed groups within the *S. erinaceieuropaei* complex (a, b, and c).

To genetically characterize the parasite in the Australian isolate Spiro_Aus1 from a dog (Figure 1; identified as *Spirometra* haplotype SH2; Section 4.1), we initially assembled the complete mitochondrial genome (Figure 2A) and then compared it with data available in public databases; its sequence was the same as that with GenBank accession no. KU852381.1 (https://www.ncbi.nlm.nih.gov/nuccore/KU852381.1; accessed 10 May 2025), except for synonymous and non-synonymous [Ala<–>Val] alterations at positions 1132 and 1655 (*cyt*b), respectively, and a non-synonymous [Leu<–>Phe] difference at position 6415 (*nad-3*) (Figure 2A—inset-table b). Subsequently, we undertook a phylogenetic analysis of mitochondrial protein-coding gene sequence data and revealed the relationship of Spiro_Aus1 with selected *Spirometra* taxa for which data were publicly available (Figure 2B). There was strong support (pp = 1.00) that Spiro_Aus1 clustered with *S. erinaceieuropaei/S. mansoni* lineage (group a) and moderate support (pp = 0.8 to 1; group b) for Spiro_Aus1 grouping with representatives of *Spirometra* from China and Japan, to the exclusion of group c representing the *S. erinaceieuropaei* SerJ isolate (OX421841), which was strongly supported (pp = 1.0).

### 2.2. Inference of Ploidy and Nuclear Genome Assembly

Prior to nuclear genomic assembly, we assessed the ploidy of the parasite using short-read data derived from total genomic DNA (Table S1) and showed (using a 21-mer setting) that coverage-peaks were consistent with a triploid organism (AAA; 94.5%) with a genome size of ~612 Mb (Figure S1). Subsequently, the long-read contigs and Hi-C sequence data (131 Gb, ~100-fold; Table S1) produced a nuclear genome assembly of ~1.73 Gb for Spiro_Aus1 (scaffold N50 = 68.1; scaffold L50 = 11; proportion of gaps = 0.02%; Table 1). This assembly contained 315 contiguous sequences, compared with 5723 and 7388 in previous assemblies of related taxa [8]. Most aligned, unique Hi-C reads inferred 18 million ‘inter-chromosomal’ and 144 million ‘intra-chromosomal’ contacts (Table S2). The benchmarking universal single-copy orthologs (BUSCO) metrics inferred increased genome completeness (68.2%) compared with previous assemblies (62.8% or 63.4%) (Table 1).

### 2.3. Inference of Nine Chromosomes

**Figure 3:**
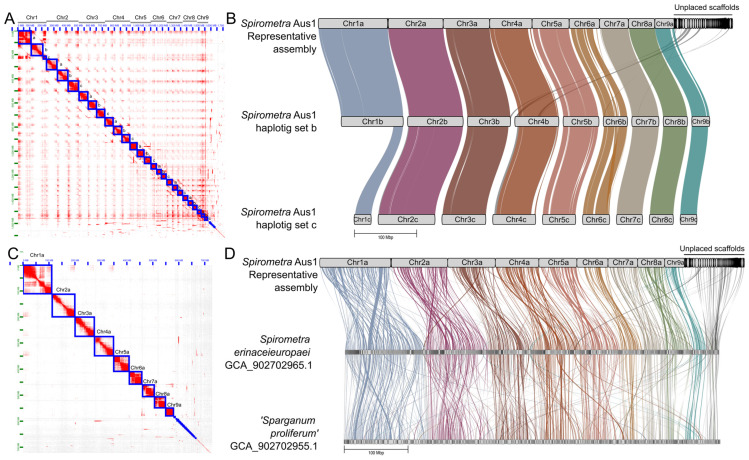
The triploid genome of *Spirometra* (Spiro_Aus1), and synteny comparisons. Panel (**A**): Hi-C matrix of the spatial clustering of Hi-C reads to three sets of nine chromosomes (Ch1a-c to Ch9a-c) and unplaced scaffolds. Panel (**B**): Synteny and contiguity of the representative assembly (designated RepA) of Spiro_Aus1 (i.e., Chr1a to Chr9a plus unplaced scaffolds) with respect to the phased haplotigs (Chr1b to Chr9b, and Chr1c to Chr9c). Chromosomes plus unplaced scaffolds aligned in 23 syntenic blocks to the ChrA of Spiro_Aus1 (haplotig sets b and c). Panel (**C**): Hi-C matrix of the spatial clustering of Hi-C reads to the nine representative chromosomes (Ch1a to Ch9a) and unplaced scaffolds in RepA of Spiro_Aus1. Panel (**D**): Synteny and contiguity of RepA of Spiro_Aus1 with respect to the draft genomes of *Spirometra erinaceieuropaei* and *Sparganum proliferum* [8]. Scaffolds are arranged in linear GENESCAPE plots, with nine chromosomes inferred for Spiro*_*Aus1, and syntenic blocks linking regions in the three genomes representing *Spirometra*. RepA of Spiro_Aus1 aligned in 451 and 403 syntenic blocks to *S. erinaceieuropaei* and *Sparganum proliferum*, respectively.

The final nuclear genomic assembly of Spiro_Aus1 comprised 27 phased chromosome-length scaffolds containing 91% of all assembled scaffolds (Figure 3A). The relative sizes of the nine chromosomes were consistent with the known karyotype of *Spirometra* [23]. This final assembly was curated and presented as follows: (i) a complete assembly (ComA): all scaffolds, including the phased haplotigs (a, b, and c) of all chromosome-length scaffolds; (ii) a representative assembly (RepA) comprising nine chromosome-length scaffolds (haplotig set a) and unplaced scaffolds; and (iii) the assembly representing all nine chromosomes (ChrA; haplotig set a) without unplaced scaffolds (see Table 1).

Assembly ComA had chromosome-length scaffolds varying from 111.6 Mb (Chr1a) to 27.0 Mb (Chr9c) (Table 1). Assembly RepA was 728.6 Mb in size (N50 = 59.8 Mb; L50 = 5) and comprised 9 chromosome-length scaffolds and 288 unplaced scaffolds with few or ambiguous contacts (preventing their placement within the phased chromosome assembly); 60 of these unplaced scaffolds were >1 Mb in length and most (>60%) were >100 kb in length (Figure 3). ChrA comprised 572 Mb (N50 = 68.1 Mb and L50 = 4). The removal of chromosome haplotig sets b and c (Figure 3) improved the number of inferred BUSCO single-copy genes (from 5.1% to 59.6%) and reduced the BUSCO complete- and duplicated-gene score from 63.1% to 6.3% (Table 1). For ChrA, the complete BUSCO scores reduced slightly from 68.2% to 63.8%, but further improved the BUSCO score estimate to 63.3% and reduced duplicates to 0.5%.

### 2.4. Nuclear Genome Annotation

More than half (53.2%) of assembly ComA of the Spiro_Aus1 genome was repetitive (Table S3) and included transposable elements (1.4% DNA transposons and 27.4% retrotransposons). Half of DNA transposons were classified as Tc1-IS630-Pogo-like. Retrotransposons were mainly long interspersed nuclear elements (LINEs; 23.7%) and long terminal repeats (LTR; 2.7%). The remaining repeat content included unclassified (24.4%) or simple (1.3%) repeat elements (Table S3).

Transcriptomic data for Spiro_Aus1 (Table S1) and protein data predicted for *S. erinaceieuropaei* or *Sparganum proliferum* [8] were used as evidence to support protein-coding genes in ComA of the Spiro_Aus1 genome. A total of 27,172 genes were predicted from repeat-masked assembly ComA and then annotated (Table 2 and Table 3). Following the removal of chromosome-length haplotigs b and c, 11,064 genes were predicted, with most genes (*n* = 9314) represented in the 9 chromosomes (ChrA). Despite using the protein models as evidence, fewer (49–56%) protein-coding genes were inferred for Spiro_Aus1 than for previously published genome assemblies for distinct genotypes of *Spirometra* [8].

Other statistics for annotated genes were similar: i.e., mean gene length (14,342 vs. 15,539 to 16,017), mRNAs (1479 vs. 1361 to 1395 bp), exons (224 vs. 225 to 234 bp) and introns (2400 vs. 2795 to 2925 bp); mean protein length (495 vs. 401 to 407 amino acid [aa] residues) (Table 2). Within the predicted gene set, we identified 618 (64.8%) of 954 complete, conserved metazoan genes by BUSCO analysis, consistent with metrics for gene sets of other flatworms [16,17], suggesting that this gene set (for ComA) represents most of the nuclear genome. The selection of a representative genome assembly (i.e., RepA) removed most BUSCO duplications, with a slight decrease (4.8%) in the number/percentage of predicted complete BUSCO genes (Table 2).

**Figure 4:**
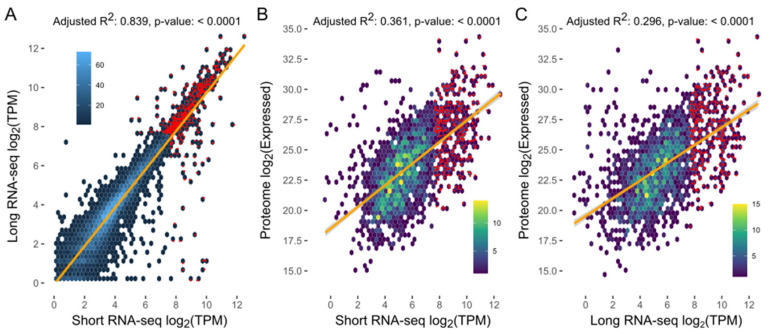
Correlations between levels of transcription (transcripts per million, TPM) for long reads vs. short reads by RNA-sequencing, and between levels of transcription (long-read or short-read RNA-seq data) and expression of protein genes encoded in the representative genome (RepA) of *Spirometra* (Spiro_Aus1). Adjusted R^2^ values and *p*-values indicated at the top of individual panels.

The short- and long-read transcriptomes assembled for Spiro_Aus1 provided support for ~78% and ~72% of protein-coding, respectively (with TPM [=transcripts per million] of ≥0.5) (Table 3; Table S4), with ~70% of genes having transcriptional support using both data sets, and displaying a direct association between RNA short-read and long-read TPM values (adjusted R^2^: 0.838; *p*-value: <0.0001; Table 3; Figure 4A). The proteome of Spiro_Aus1 provided support for ~20% of protein-coding genes, most of which also had transcriptional support (Table 3). There was also a direct association between protein expression and levels of transcription (i.e., TPM) using short-read RNA (adjusted R^2^: 0.361; *p*-value: < 0.0001; Figure 4B) and long-read data (adjusted R^2^: 0.296; *p*-value: <0.0001; Figure 4C).

Subsequently, we functionally annotated 77.7% of protein-coding genes (*n* = 21,119) using information from the eggNOG (*n* = 20,193; 74.3%) and InterProScan (*n* = 20,005; 73.6%) databases (Table 3; Tables S5 and S6). Functional Pfam domains and GO terms were assigned to 17,883 (65.6%) and 15,032 (55.3%) genes, respectively. More than half of the encoded genes (58.6%; *n* = 15,925) had protein homology to those in the KEGG database with an assigned orthology (KO) term (Table 3; Table S5). InterProScan-linked Reactome and MetaCyc pathway information was also inferred for 18,323 (67.4%) and 14,043 (51.7%) of genes, respectively (Table 3; Table S6). Proportions of annotated genes in assemblies ComA and RepA of the Spiro_Aus1 genome were relatively consistent with most annotated genes present in chromosome-length scaffolds (Table 3).

### 2.5. Synteny

Upon pairwise comparison, there was a marked synteny between the chromosomal haplotigs of the Spiro_Aus1 genome and the published nuclear genomes for *S. erinaceieuropaei* and *Sparganum proliferum* (Figure 3; Table 4). Most synteny was between assembly RepA and the chromosome-length haplotig set b (ChrB) for Spiro*_*Aus1 (Figure 3B; Table 4), with 100% (538 Mb; *n* = 9) of scaffolds in the latter aligning to 84.1% (613 Mb; *n* = 18) of the former in 23 syntenic blocks of 7106 orthologous gene pairs. Synteny was similar between assembly RepA and chromosome-length haplotig set c (ChrC) of the triploid Spiro_Aus1 genome (Figure 3B; Table 4), with 23 syntenic blocks of 5746 orthologous gene pairs (the reduced number being due to a short chromosome 1c-scaffold) (Figure 3B). Although less synteny was seen among previously published genomes for *Spirometra* taxa (Figure 3D; Table 4), most synteny was between RepA of the Spiro*_*Aus1 genome and the *Sparganum proliferum* genome [8], with 72.5% (474 Mb; *n* = 333) of scaffolds in the latter aligning to 94.1% (686 Mb; *n* = 43) of the former in 403 syntenic blocks of 6248 orthologous gene pairs. The least synteny was seen between RepA of Spiro_Aus1 and *S. erinaceieuropaei*, with 56.1% (446 Mb; *n* = 395) of scaffolds in the latter aligning to 91.7% (668 Mb; *n* = 38) of the former in 451 syntenic blocks of 5197 orthologous gene pairs. Unplaced scaffolds in RepA comprised regions (and associated orthologous genes) that were syntenic to both *S. erinaceieuropaei* (25 scaffolds) and *Sparganum proliferum* (20 scaffolds) (Figure 3D). These unplaced scaffolds lacked long-read or Hi-C contacts as support for their placement within any of the nine chromosome-length haplotigs (Figure 3).

Interestingly, there was a total of 432 highly transcribed (>200 TPM) protein-coding genes in spargana (Spiro_Aus1) (Figure 4; Table S4), 321 of which were linked to 307 KEGG terms. Enriched protein groups representing highly transcribed genes were linked to exosomes (*n* = 74 KO-terms), ribosome (61), membrane trafficking (46), cytoskeleton proteins (28), chaperones and folding catalysts (27), and messenger RNA biogenesis (20) (Table 5).

As excretory/secretory (ES) proteins are recognized to play central roles in host-parasite interactions [24,25], we explored the nature and extent of these proteins encoded in the gene sets of Spiro_Aus1; ~5% of each gene set defined (e.g., 565 genes in RepA) were predicted to encode extracellular ES proteins, based on the presence of a signal peptide domain (8.2% of each gene set) and the absence of one or more transmembrane domains (~18.5% of each gene set) (Table 3; Table S4). Using both transcriptomic and proteomic data, there was evidence for active transcription and translation of 146 ES protein-coding genes in RepA of the Spiro_Aus1 genome. Within this secretome, 66 proteins were assigned 75 KEGG terms and represented protein groups including chaperones and folding catalysts (19 KO-terms), exosome (15), membrane trafficking (12), and lectins (4) (Table 5). Of these 146 transcribed and translated ES proteins, 50 were orphans, with no functional annotation inferred using data or information in the eggNOG or InterProScan databases (Table S4).

## 3. Discussion

Despite advances in parasite genomics, many published draft genomes of helminths, including those of socioeconomically important flatworms (see WormBase ParaSite), remain incomplete [26]. Here, we employed long-read and in situ Hi-C data sets, together with available short-read data, to assemble the first complete, triploid genome for a member of the *Spirometra* complex from Australasia. We believe that this first chromosome-contiguous nuclear genome for this neglected zoonotic parasite should provide an invaluable resource to enable research of the systematics, the molecular biology, and the biochemistry of members of the *Spirometra* complex, as well as the pathogenesis of proliferative sparganosis.

Published works have indicated significant genetic complexity and variability within the genus *Spirometra* (reviewed by [8]). Over the years, 60 ‘species’ of *Spirometra* have been reported [27,28], but a recent review scrutinized all published information and provided some new molecular evidence for the existence of four valid species and six (genetic) lineages [8]. However, there may be a limitation in proposing a systematic framework for *Spirometra* based solely on a phylogenetic analysis of cytochrome *c* oxidase subunit 1 gene (*cox*1) or mitochondrial genomic sequence data. Nonetheless, the present reference nuclear genome should provide a sound starting point to now rigorously test the hypothesis regarding the species composition of the *Spirometra* complex using a phylogenetic approach; to assess levels of genetic variability both within and among valid species; to genetically characterize taxa that might emerge as cryptic species; and to define a panel of genetic markers to accurately identify and distinguish species for diagnostic purposes. As a next step, we would propose that a range of (preferably karyotyped) samples (with associated voucher specimens) representing all four valid species and the yet undescribed species (*Spirometra* sp. 1) from diverse geographic locations (e.g., Africa, Central Europe, Asia, Australasia, and the Americas) be subjected to whole nuclear genome sequencing and assembly (using the same or a similar approach as used here), followed by genomic comparisons and subsequent quantitation of genetic variability. We believe that it would be advantageous to combine cytogenetic analyses of *Spirometra* samples (cf. [23]) with comparative genomic investigations.

As the clinical diagnosis of human sparganosis—particularly the proliferative form—is challenging, PCR-based or short-read DNA sequencing methods (e.g., Illumina), combined with computer tomography (CT) imaging, can assist in making a definitive diagnosis. Conventional PCR-based sequencing of *cox*1 is useful as complementary tool but may not allow an accurate classification (to species or genotype) due to the use of relatively short and select mitochondrial gene regions (~400–600 bp) for ‘barcoding’ [29,30]. Short-read sequencing of genomic DNA isolated from biopsy material and/or plerocercoids is an effective diagnostic approach, but it is more expensive than PCR-based sequencing, and sequence data may not allow a test sample to be precisely classified to the genotype level in the absence of a panel of complete mitochondrial and nuclear genomes representing currently (proposed) recognized species [8]. This is another reason why assembling a panel of reference genomes will be of major practical use in a clinical context. Such a panel would also provide a basis for meaningful molecular epidemiological, phylogenomic, and population genetic investigations of *Spirometra* at different levels (both regional and global), bearing in mind that it would be beneficial to also study the ploidy of parasites in individual geographical origins. Triploidy in the present *Spirometra* sample (i.e., Spiro_Aus1) linked to a fatal sparganosis case begs the question as to whether there might be a relationship between ploidy and pathogenicity. Moreover, epidemiological tracking should be particularly valuable in defining/establishing precise transmission patterns (and life cycles) for particular species and genotypes of *Spirometra*.

Looking ahead, mRNA and/or 5′-end RNA sequencing [31] of multiple developmental stages (including coracidium, procercoid, plerocercoid, and adult), together with long-read RNA-sequencing, should assist in the enhanced curation of gene models. Additional efforts are also needed to annotate currently unknown genes using orthology-based assignment [32,33], providing an avenue for comprehensive studies of gene transcription, expression, and regulation in this cestode. For instance, establishing the interactions/relationships of novel, full-length mRNAs with non-coding RNAs in *Spirometra* would be very informative. Logically expanding such work, this new nuclear genomic resource for *Spirometra* should also enable combined transcriptomic, proteomic, and metabolomic (multi-omic) explorations, to gain an understanding of critical biological pathways and processes in *Spirometra*, and, importantly, its interactions with the host animal. Stimulated by research findings from early studies of spargana of a particular taxon (*S. mansonoides*, now proposed to be within the *S. decipiens* complex; ref. [8]), another interesting area to pursue would be to explore the molecular and biochemical roles of the sparganum growth factor (SGF) [34,35].

The new nuclear genome reported here could also support functional genomics work on different developmental stages of *Spirometra*, particularly plerocercoids that cause sparganosis. Given that gene-specific knockdown by RNA interference (RNAi) [36,37,38] and CRISPR/Cas9 systems [39,40,41] have been shown to work in some flatworm species, there would be a unique prospect of establishing a functional genomic platform using, for instance, the Kawasaki triploid clone (Kt) of *S. erinaceieuropaei* [23]. Functional work could extend to in vivo experimentation in animals using the complete life cycle established for this parasite in order to verify the phenotypic outcome of gene knockdown. Assessing the functions of genes predicted to be essential for development, growth, and reproduction using machine learning-coupled approaches [42] in this platform could enable the discovery of *Spirometra*-selective molecular targets for the subsequent design of one or more cestocides to treat proliferative sparganosis in humans and companion animals (dogs and cats). Such a focus is important, particularly given that the treatment of this disease with current compounds, such as praziquantel, albendazole, and/or mebendazole, is not consistently effective in humans, even at very high dosages (cf. [1,43]).

While the present investigation focused on the genome of a *Spirometra* isolate from Australia (Spiro_Aus1), the utility of our sequencing–informatic methodology to complete a relatively large genome (≥600 Mb) at modest expense (USD 2000) highlights a cost-effective and broadly applicable approach for investigating a range of cestode parasites of medical and veterinary importance. We hope that this high-quality nuclear genome will accelerate both fundamental and translational research on sparganosis, and support the discovery of new targets for intervention against this neglected disease in humans and companion animals. Importantly, this work provides a foundation for the systematic genomic exploration of the genus *Spirometra*, enabling comparative analyses across species and lineages to resolve taxonomic uncertainties, clarify evolutionary relationships, and identify lineage-specific biological and pathogenic traits.

## 4. Materials and Methods

### 4.1. Parasite Material

Structures that appeared to be macroscopically consistent with spargana (plerocercoids = larvae) were collected by one of the authors (A.B.) during exploratory surgery from the abdominal cavity of a dog (a spayed female Labrador; 5 years of age) suffering from proliferative sparganosis (Figure 1A) in Perth, Western Australia, then frozen (−80 °C) in 80 µL aliquots or preserved in RNAlater (Thermofisher, Waltham, MA, USA). The structures in this isolate (designated Spiro_Aus1) were examined by conventional light microscopy [21] and were morphologically/histologically consistent with spargana (Figure 1B). PCR-based sequencing from genomic DNA revealed a cytochrome *c* subunit 1 (*cox*1; 393 bp) sequence with a perfect match to haplotype SH2 (Figure 2A—inset-table a; ref. [22]).

### 4.2. Isolation of Genomic DNA, and Construction and Sequencing of DNA Long Read, Short-Read and In Situ Hi-C Libraries

High-quality genomic DNA was isolated from ~50 mg of spargana using the Nanobind Tissue Big DNA kit, according to the manufacturer’s instructions (Circulomics, Baltimore, MD, USA). The integrity of the DNA was verified using an Agilent 4200 TapeStation system (ThermoFisher) and using Genomic DNA ScreenTape (ThermoFisher).

First, a short-insert (500 bp) genomic DNA library was constructed from spargana DNA (1 µg) and paired-end sequenced (150 base reads) using TruSeq chemistry and the NovaSeq sequencing platform (Illumina, San Diego, CA, USA). The sequence reads produced were verified, and low-quality sequences (<Phred 25) and adaptors were removed using fastp v0.23.2 (https://bio.tools/fastp). Second, a HiFi SMRTbell library was constructed using the SMRTbell Express Template Prep Kit v2.0 (PacBio) according to the manufacturer’s instructions. High-molecular-weight genomic DNA was sheared to a size range between 15–20 kb, and a HiFi SMRTbell library was constructed and then sequenced using Sequel II chemistry 2.0 in a SMRT cell on a PacBio Sequel II sequencer (PacBio, Menlo Park, CA, USA). Third, in situ Hi-C sequencing was performed using the Arima-HiC+ for high coverage kit, according to manufacturer’s instructions (Arima Genomics, Carlsbad, CA, USA). The high-molecular-weight DNA from ~100 mg of packed spargana was restriction-digested using the Arima enzyme mix (see manufacturer’s instructions). The indexed Arima Hi-C DNA library was then sequenced using the NovaSeq platform.

### 4.3. Isolation of Total RNA and Construction and Sequencing of Long-Read and Short-Read Libraries

RNA was isolated from ~50 mg of RNAlater-preserved spargana using the TriPure Isolation Reagent (Sigma Aldrich, Burlington, MA, USA) and DNase-treated using a TURBO DNA-free^TM^ kit (Thermo Fisher Scientific). The size, integrity (RNA integrity number, RIN), and concentration of RNA were determined using a 4200 TapeStation System RNA ScreenTape Assay (Agilent Technologies, Waldbronn, Germany) and a Qubit^®^ 3.0 flourometer RNA High Sensitivity Assay (Life Technologies, Carlsbad, California, USA).

A TruSeq Stranded mRNA (Illumina) short-read library (150 bp, paired-end) was prepared from the total RNA according to the manufacturer’s instructions and sequenced on an Illumina NextSeq 500 sequencer. The paired short RNA-seq reads produced were assessed for quality, and low-quality (<Phred 25) sequence reads and adaptors were removed using fastp v0.23.2 (https://bio.tools/fastp). Messenger RNA (mRNA) was purified from total RNA using the Dynabeads^®^ mRNA Purification Kit (Thermo Fisher Scientific). In addition, a long-read library was prepared from the same total RNA using the Oxford Nanopore direct RNA-sequencing kit (SQK-RNA002; Oxford Nanopore Technologies, Oxford, UK), according to the manufacturer’s instructions. The prepared library was sequenced on a MinION sequencer (Oxford Nanopore Technologies) for 48 h until no more active pores were available, using an EXP-FLP002 flow cell priming kit and a R9.4.1 flow cell (FLO-MIN106). Following sequencing, bases were ‘called’ from raw FAST5 reads using the Guppy v6.3.8 program (Oxford Nanopore Technologies) with the ‘rna_r9.4.1_70bps_hac.cfg’ model and stored in the FASTQ format.

### 4.4. Assessing Genome Size, Heterozygosity and Ploidy

The short-insert (500 bp) genomic DNA library representing spargana (isolate Spiro_Aus1) was quality-filtered (retain > Phred 25), adapters were removed using the fastp v0.23.2 program [44], and genome size, heterozygosity, and ploidy were estimated using the GenomeScope v2.0 and smudgeplot v0.2.4 packages [45]. Input into each program was the frequency of 21-mers in the raw short-read data, determined using kmc v3.1.1 [46]. GenomeScope analyses were performed assuming a diploid or triploid genome model (based on some prior evidence for *Spirometra* from the literature; ref. [23]).

### 4.5. Assembly and Scaffolding of Genomic Contigs and Removal of Potential Contaminants

The mitochondrial and nuclear genomes representing Spiro_Aus1 were assembled using the following methodology: PacBio long-reads from the genomic DNA were used to assemble contigs employing CANU v2 (https://canu.readthedocs.io/en/latest/; accessed 5 December 2024) with the –pacbio-hifi option and a genome size estimate of 680 Mb. Scaffolds were combined with the in situ Hi-C data using YaHS v1.1 [47]. These Hi-C data were used to inspect and curate chromosomes using Juicer v1.6 [48] and Juicebox Assembly Tools v1.9.8 (http:github.com/aidenlab/Juicebox/wiki/Juicebox-Assembly-Tools/; accessed 10 December 2024) to achieve chromosome-length scaffolds. To remove possible contaminating canine or microbial host DNA, genomic scaffolds were assessed using blobtools v1.1 (https://github.com/DRL/blobtools; accessed 10 December 2024). Contigs with similarity to chordates (including dog) or taxa external to cestodes were removed from the assembly. An assessment of completeness (in genome-mode) was conducted using BUSCO v5.4.4 using the default MetaEuk gene predictor (https://busco.ezlab.org/busco_userguide.html; ref. [49]; accessed 15 December 2024).

### 4.6. Gene Models and Annotation

Repeat elements in the nuclear genome for Spiro_Aus1 were predicted using RepeatModeler v2.0.4 (https://github.com/Dfam-consortium/RepeatModeler; accessed 1 January 2025; ref. [50]). Protein families incorrectly reported as repeats were removed, guided by the protein annotation of a six-frame translation of the repeat library using eggNOG-mapper v5 [51]. The final repeat element library was used to establish the repeat content of each genome data set and to softmask the complete assembly using RepeatMasker v4.1.4 [52]. Gene models were predicted using BRAKER v3 (https://github.com/Gaius-Augustus/BRAKER; accessed 15 January 2025) using long- and short-read RNA-seq data generated from Spiro_Aus1 spargana (Table S1). First, short-insert RNA-seq reads were quality-filtered (retain > Phred 25), and adapters were removed using the fastp v0.23.2 progrem [44]; the reads were then k-mer-corrected using Rcorrector [53] (Song and Florea, 2015). Filtered reads were mapped to the complete genome using HISAT v2.1.0 [54] and used to assemble a transcriptome with TRINITY v2.8.5 (https://apolo-docs.readthedocs.io/en/latest/software/applications/trinity/trinity-2.8.5/index.html; accessed 20 January 2025) using a genome-guided approach. Coding regions were inferred using codAn v1.2 [55] and redundancy-removed using cd-hit-est v4.8.1 (https://sites.google.com/view/cd-hit; accessed 22 January 2025). Then, to infer transcripts, long-reads were mapped to the complete genome using minimap2 v2.17 [56], employing the options *-ax splice*, *-uf*, and *-k14*. Subsequently, the program FLAIR v1.6.1 [57] was employed to correct splice junctions created by mapped long-reads using high-quality, mapped short-reads and to ‘collapse’ mapped long-reads into transcripts using the “--stringent” option. Next, BRAKER was run in ‘etp’ mode by using transcriptomic (short- and long-read transcripts, and mapped short-read RNA-seq from Spiro_Aus1 spargana) and protein models inferred for *Spirometra erinaceieuropaei* and *Sparganum proliferum* [8].

The completeness of the gene set was assessed (in protein-mode) using the tool BUSCO v5.4.4 [49]. The annotation of each inferred amino acid sequence was achieved using InterPro v5.35 [58] and eggNOG-mapper v5.0 [33]. Protein groups and pathways were inferred based on homology to KEGG orthology (KO) terms linked to curated KEGG BRITE and pathway hierarchies. Signal peptide domains were predicted using SignalP v6.0 [59] and transmembrane domains employing TMHMM v2 [60]. Evidence of gene transcription was inferred by mapping short and long RNA-seq data to the genome using HISAT2 v2.1.0 [54], and the level of transcription per gene (in ‘transcripts per million’, TPM) was inferred using StringTie v2.1.2 [61]. Gene models were inferred to have transcriptional support if one or more libraries had a TPM value of >0.5. Gene models predicted from Spiro_Aus1 were deposited in the National Center for Biotechnology Information (NCBI) database (accession no. PRJNA1104264; JBEVYL000000000).

### 4.7. Comparative Mitochondrial Genomic Analyses

Mitochondrial protein-coding genes of *Spirometra* (isolate Spiro_Aus1) were compared with those available in NCBI for members of the genus *Spirometra* and *Sparganum proliferum* [8]. Nucleotide sequences were aligned using the MUSCLE program [62]. The optimal substitution model for each aligned sequence was then assessed using the ModelTest-NG v0.1.6 program [63]. The aligned sequences were then subjected to phylogenetic analysis using the Bayesian inference (BI) tree-building method, employing Monte Carlo Markov chain analysis in the MrBayes v3.2 program [64]. The posterior probabilities (pp) were calculated using the selected substitution model, generating 3,000,000 trees and sampling every 200th tree until the potential scale reduction factors for each parameter approached 1. The initial 25% of trees were discarded as burn-in, and the others were used to construct a majority rule tree. Trees were rendered and annotated using ggtree [65] in the R language.

### 4.8. Proteomic Analysis

The somatic proteome of *Spirometra* (isolate Spiro_Aus1) was analyzed using an established protocol [66]. In brief, four samples of spargana (100 µL each) were individually washed five times with physiological saline (4 °C), pelleted, suspended in lysis buffer (8 M urea in 100 mM triethyl ammonium bicarbonate, pH 8.5), and ultrasonicated on ice (eight sonication cycles: 30 s on–30 s off at 20 kHz). Each sample was supplemented with 10 µL of protease inhibitors (cocktail set I, Merck, Denmark) and incubated at 23 °C for 30 min. Then, samples were centrifuged at 15,000 × *g* for 20 min at 4 °C, and individual supernatants collected. Protein concentrations were measured using a bicinchoninic acid (BCA) protein assay kit (Thermo Fisher Scientific, Waltham, MA, USA). Each protein sample (100 μg) was reduced, alkylated, and digested (Lys-C/trypsin Mix; Promega, Madison, WI, USA), and the peptides were acidified and purified using Oasis HLB cartridges (Waters, Milford, MA, USA). Tryptic peptides were analyzed using Fusion Lumos Orbitrap mass spectrometers (Thermo Fisher, Waltham, MA, USA) using an established protocol [67]. The proteome predicted for Spiro_Aus1 was used for protein identification using MaxQuant [68]. Peptides were accepted based on a false discovery rate (FDR) of <0.01 at both the peptide and protein levels. Only proteins with ≥2 unique peptides, detected in ≥3 replicates, were accepted. Proteomic data are available (accession no. PXD040667) via the PRoteomic IDEntification (PRIDE) database (https://www.ebi.ac.uk/pride/).

## Figures and Tables

**Table 1:** Features of the reference genome for *Spirometra* (Spiro_Aus1; three distinct assemblies) from a dog with suspected proliferative sparganosis compared with previous assemblies from *Spirometra erinaceieuropaei* from a human patient of Chinese ethnicity with long-term cerebral sparganosis in the UK (cf. [7]) and *Sparganum proliferum* from BALB/c mice infected with spargana originally isolated from a Venezuelan patient in 1981, and from a Japanese four-lined ratsnake (*Elaphe quadrivirgata*) in 2014 [8].

Assembly	Complete Assembly [ComA] ^a^	Representative Assembly [RepA] ^a^	Chromosomal Assembly [ChrA] ^a^	*Spirometra* *erinaceieuropaei* ^b^ GCA_902702965.1	*Sparganum* *proliferum* ^b^ GCA_902702955.1
Number of scaffolds	315	297	9	5723	7388
Total size of scaffolds	1,730,282,878	728,638,509	572,004,676	796,029,360	653,387,223
Longest scaffold	111,593,610	111,593,610	111,593,610	5,490,141	8,099,213
Number of scaffolds: >100 K; 1 M; 10 M	198; 78; 27	180; 60; 9	9; 9; 9	1304; 200; 0	724; 203; 0
N50 scaffold length; L50 scaffold count	68,120,031; 11	59,837,588; 5	68,120,031; 4	820,922; 271	1,241,503; 146
Scaffold GC (%)	45.4	45.4	45.4	40.7	41.8
Scaffold N (%)	0.02	0.02	0.02	9.8	7.8
Number of contigs	1793	854	495	50,415	41,218
Longest contig	11,343,096	11,343,096	11,343,096	471,202	304,998
Number of contigs: >100 K; 1 M; 10 M	1488; 591; 1	661; 256; 1	443; 204; 1	465; 0; 0	451; 0; 0
N50 contig length; L50 contig count	1,895,074; 271	1,793,361; 120	2,026,377; 85	29,662; 6600	32,650; 5100
Contig GC (%)	45.4	45.4	45.4	45.1	45.3
Genome completeness and accuracy:					
Complete BUSCO ^c^	651 (68.2%)	629 (65.9%)	609 (63.8%)	599 (62.8%)	605 (63.4%)
Complete single-copy BUSCO	49 (5.1%)	569 (59.6%)	604 (63.3%)	529 (55.5%)	584 (61.2%)
Complete and duplicated BUSCO	602 (63.1%)	60 (6.3%)	5 (0.5%)	70 (7.3%)	21 (2.2%)
Fragmented BUSCO	70 (7.3%)	78 (8.2%)	74 (7.8%)	100 (10.5%)	97 (10.2%)

^a^ ‘Complete assembly (ComA)’ includes all three sets of haplotigs (a, b, and c) of the triploid genome (Spiro_Aus1); representative assembly (RepA) includes all scaffolds linked to the nine chromosomes and unplaced scaffolds; ‘chromosomal assembly (ChrA)’ includes only scaffolds linked to all nine chromosomes. ^b^ Kikuchi et al. [8]. ^c^ Number of benchmarking universal single-copy orthologs (BUSCO) identified (genome-mode), and percentage of the 954 genes within the Metazoa Odb10 dataset (https://busco.ezlab.org/frames/euka.htm; accessed 12 April 2025).

**Table 2:** Features of the gene sets for a reference genome (Spiro_Aus1; three distinct assemblies) for *Spirometra* from a dog with proliferative sparganosis compared with previous assemblies from *Spirometra erinaceieuropaei* from a human patient of Chinese ethnicity with long-term cerebral sparganosis in the UK [7], and *Sparganum proliferum* from BALB/c mice infected with spargana originally isolated from a Venezuelan patient in 1981, and from a Japanese four-lined ratsnake (*Elaphe quadrivirgata*) in 2014 [8].

Features	Complete Assembly [ComA] ^a^	Representative Assembly [RepA] ^a^	Chromosomal Assembly [ChrA] ^a^	*Spirometra* *erinaceieuropaei*^b^ GCA_902702965.1	*Sparganum* *proliferum*^b^ GCA_902702955.1
Numbers of genes (mRNAs)	27,172 (31,299)	11,064 (12,750)	9314 (10,734)	20,774 (20,774)	16,508 (16,508)
Gene length ^c^	14,596 ± 19,177	14,342 ± 18,657	14,654 ± 18,839	15,539 ± 20,390	16,017 ± 21,161
mRNA length	1490 ± 1400	1479 ± 1384	1504 ± 1414	1361 ± 1338	1395 ± 1422
Coding domain length	1489 ± 1397	1476 ± 1377	1504 ± 1414	1361 ± 1338	1395 ± 1422
Number of exons	6.6 ± 6.3	6.6 ± 6.2	6.7 ± 6.3	5.8 ± 5.5	6.2 ± 5.9
Exon length	222.3 ± 298.5	224.3 ± 302.2	222.7 ± 301.6	234.4 ± 290.0	225.8 ± 274.3
Intron length	2400 ± 3599	2388 ± 3576	2376 ± 3582	2925 ± 4573	2797 ± 4396
Protein length	495.3 ± 465.7	491.1 ± 459.1	500.4 ± 471.2	407.5 ± 405.9	401.5 ± 425.1
Completeness:					
Complete BUSCO ^d^	618 (64.8%)	572 (60.0%)	549 (57.6%)	617 (64.7%)	560 (58.7%)
Complete single-copy BUSCO	75 (7.9%)	510 (53.5%)	534 (56.0%)	557 (58.4%)	542 (56.8%)
Complete and duplicated BUSCO	543 (56.9%)	62 (6.5%)	15 (1.6%)	60(6.3%)	18 (1.9%)
Fragmented BUSCO	70 (7.3%)	72 (7.5%)	72 (7.5%)	87 (9.1%)	110 (11.5%)

^a^ ‘Complete assembly’ (ComA) includes the three haplotig sets a, b, and c (cf. Figure 3) of the triploid genome for *Spirometra* (Spiro_Aus1); ‘Representative assembly’ (RepA) includes all scaffolds linked to all nine chromosomes and all unplaced scaffolds; ‘Chromosomal assembly’ (ChrA) includes only scaffolds linked to all nine chromosomes. ^b^ Kikuchi et al. [8]. ^c^ Lengths are in base pairs (bp) or amino acids (mean ± standard deviation). ^d^ Number of benchmarking universal single-copy orthologs (BUSCO) identified (protein-mode), and percentage of the 954 genes within the Metazoa_Odb10 dataset (https://busco.ezlab.org/frames/euka.htm; accessed 10 April 2025).

**Table 3:** Annotation of the gene models for the triploid reference genome (Spiro_Aus1; three assemblies) for *Spirometra* from a dog with proliferative sparganosis.

Description	Complete Assembly [ComA] ^a^ (%)	Representative Assembly [RepA] ^a^ (%)	Chromosomal Assembly [ChrA] ^a^ (%)
Number of genes	27,172	11,064	9314
Evidence (short-read transcripts) ^b,c^	21,061 (77.5)	8577 (77.5)	7289 (78.3)
Evidence (long-read transcripts) ^c^	19,361 (71.3)	7927 (71.7)	6801 (73.0)
Evidence (both short- and long-read transcripts) ^c^	18,890 (69.5)	7748 (70.0)	6643 (71.3)
Evidence (protein expression)	5549 (20.4)	2223 (20.1)	1921 (20.6)
Evidence (transcription and expression) ^d^	5482 (20.2)	2195 (19.8)	1901 (20.4)
*Homology searches*			
eggNOG mapper ^b^	20,193 (74.3)	8289 (74.9)	6969 (74.8)
InterProScan domains	20,005 (73.6)	8183 (74.0)	6904 (74.1)
PFAM domains	17,833 (65.6)	7293 (65.9)	6154 (66.1)
Gene ontology (GO) results ^f^	15,032 (55.3)	6083 (55.0)	5180 (55.6)
KEGG orthologues	15,925 (58.6)	6456 (58.4)	5474 (58.8)
Reactome pathways	18,323 (67.4)	7491 (67.7)	6326 (67.9)
MetaCyc pathways	14,043 (51.7)	5711 (51.6)	4848 (52.1)
Proteins with signal peptides	2239 (8.2)	902 (8.2)	762 (8.2)
Transmembrane (TM) domains	5018 (18.5)	2040 (18.4)	1743 (18.7)
Excretory/secretory (ES) proteins ^e^	1405 (5.2)	565 (5.1)	470 (5.0)
Transcribed ES protein genes	1075 (3.6)	433 (3.9)	363 (3.9)
Transcribed and expressed ES protein genes ^d^	400 (1.5)	146 (1.3)	127 (1.4)

^a^ ‘Complete (ComA) assembly’ includes all three haplotig sets a, b, and c (cf. Figure 3) of the triploid genome for *Spirometra* (Spiro_Aus1); ‘Representative (RepA) assembly’ includes all scaffolds linked to all nine chromosomes and all unplaced scaffolds; ‘Chromosomal (ChrA) assembly’ includes only scaffolds linked to all nine chromosomes. ^b^ The numbers of genes and percentages of total number of gene models. ^c^ Genes with transcription support (>0.5 transcripts per million, TPM). ^d^ Evidence of transcription in short or long read RNA-seq data. ^e^ Proteins with a signal peptide but lacking a transmembrane domain. ^f^ Gene ontology (GO) terms with linked to conserved domains (InterProScan).

**Table 4:** Pairwise genome-wide synteny comparisons of the genomes representing *Spirometra* Aus1 (i.e. RepA, ChrB, ChrC; see Table 1; Figure 3), *Spirometra erinaceieuropaei* (SerJ; GCA_902702965.1) and *Sparganum proliferum* (Spr; GCA_902702955.1).

	Syntenic Scaffolds		Length of Scaffolds with Bundled Links (Percentage of Genome Assembly)		Number of Syntenic Blocks	Number of Genes
	RepA	ChrB	RepA	ChrB		
Spiro_Aus1: RepA vs. ChrB	18	9	612,663,739 (84.1%)	538,366,406 (100%)	23	7106
	RepA	ChrC	RepA	ChrC		
Spiro_Aus1: RepA vs. ChrC	16	9	622,304,671 (85.4%)	463,277,963 (100%)	23	5746
	RepA	SerJ	RepA	*S. erinaceieuropaei*		
RepA vs. SerJ	38	395	667,935,674 (91.7%)	446,608,837 (56.1%)	451	5197
	RepA	Spr	RepA	Spr		
RepA vs. Spr	43	333	685,813,185 (94.1%)	473,742,847 (72.5%)	403	6248

**Table 5:** Protein groups inferred for the representative assembly (RepA) of the genome of *Spirometra* (Spiro_Aus1) from a dog with proliferative sparganosis. These groups represent genes that were highly transcribed (>200 transcripts per million, TPM) in spargana, or inferred excretory/secretory proteins with evidence of gene transcription and translation in spargana.

Protein Groups	Total Number of KEGG Terms	Number of KEGG Terms for Highly Transcribed Genes (*p*-Value)		Number of KEGG Terms for Expressed ES Proteins (*p*-Value)	
ko04147 Exosome	324	74	(0.0000)	15	(0.0368)
ko03011 Ribosome	117	61	(0.0000	0	
ko04131 Membrane trafficking	594	46	(0.0280)	12	(0.0000)
ko04812 Cytoskeleton proteins	187	28	(0.0000)	0	
ko03110 Chaperones and folding catalysts	124	27	(0.0000)	19	(0.0002)
ko03019 Messenger RNA biogenesis	228	20	(0.0372)	0	
ko03036 Chromosome and associated proteins	459	13	(0.0001)	0	
ko03012 Translation factors	60	11	(0.0010)	0	
ko04031 GTP-binding proteins	86	8	(0.0887)	0	
ko03009 Ribosome biogenesis	175	8	(0.0795)	0	
ko03000 Transcription factors	259	6	(0.0011)	0	
ko00536 Glycosaminoglycan binding proteins	35	6	(0.0169)	0	
ko01007 Amino acid related enzymes	33	6	(0.0132)	0	
ko01009 Protein phosphatases and associated proteins	177	5	(0.0146)	0	
ko03037 Cilium and associated proteins	131	4	(0.0407)	0	
ko00537 Glycosylphosphatidylinositol (GPI)-anchored proteins	13	3	(0.0396)	2	(0.1567)
ko04091 Lectins	16	3	(0.0635)	4	(0.0142)
ko03021 Transcription machinery	143	3	(0.0103)	0	

## Data Availability

Sequence read data are accessible via the NCBI database (accession no. PRJNA1104264), and proteomic data via the PRIDE database (accession no. PXD040667).

## Supplementary Materials

The supporting information can be downloaded at https://www.mdpi.com/article/10.3390/ijms26136417/s1.
