# Supplementary material for: Chromosome-Contiguous Reference Genome for Spirometra to Underpin Future Discovery Research
Source: Int J Mol Sci. 2025 Jul 3;26(13):6417. doi: 10.3390/ijms26136417 (PMC12249497; doi:10.3390/ijms26136417)
Supplement: Supplementary file 1 [file ijms-26-06417-s001.zip › Supplementary_Figure_S1.pdf]

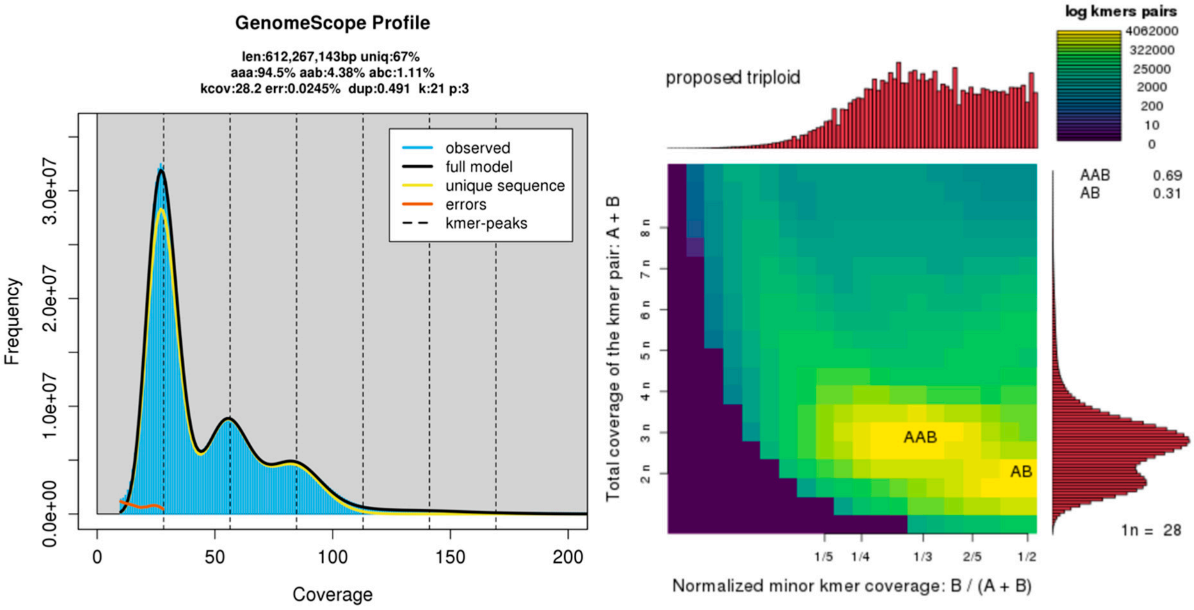

Figure S1. An assessment of ploidy for *Spirometra* (isolate Spiro_Aus1) using short-read (~500 bp) DNA data produced by Illumina sequencing. Left panel: GenomeScope2 21-mer profile, assuming a triploid genome model. Right panel: Log-transformed SmudgePlot for 21-mer paired coverage, indicating a triploidy genome for Spiro_Aus1 using Illumina data.
